# Low-dose anti-thymocyte globulin plus low-dose posttransplant cyclophosphamide as graft-versus-host disease prophylaxis in haploidentical peripheral blood stem cell transplantation combined with unrelated cord blood for patients with hematologic malignancies: a prospective, phase II study

**DOI:** 10.1038/s41409-018-0382-3

**Published:** 2018-11-16

**Authors:** Jun Yang, Jieling Jiang, Yu Cai, Su Li, Liping Wan, Jun Zhu, Huixia Liu, Shan Shao, Haitao Bai, Chun Wang, Xianmin Song

**Affiliations:** Department of Hematology, Shanghai General Hospital (affiliated to Shanghai Jiao Tong University), No. 100 Haining Road, 200080 Shanghai, China

**Keywords:** Stem-cell research, Haematological diseases

## Abstract

Nowadays, the most wildly used regimens for graft-versus-host disease (GvHD) prophylaxis in haplo-hematopoietic stem cell transplantation (Haplo-HSCT) are based on in vivo T-cell depletion (TCD) with anti-thymocyte globulin (ATG) or posttransplant cyclophosphamide (PTCy). To improve the efficiency of GvHD prophylaxis in haploidentical peripheral blood stem cell transplantation combined with unrelated cord blood (Haplo-PBSCT-Cord), a novel regimen, which is composed of low dose of ATG (5 mg/kg) and low-dose PTCy (50 mg/kg) for GvHD prophylaxis, was evaluated in a prospective phase II clinical trial (Clinicaltrials.org NCT03395860). Thirty-two patients diagnosed with hematological malignancies were enrolled in this trial. All patients received myeloablative conditioning regimens except for three patients. The cumulative incidences (CIs) of grades II-IV and III-IV acute GvHD were 19.4% (95% CI, 5.5−33.3%) and 6.9% (95% CI, 0−16.3%) by day 100, respectively. The 1-year probability of relapse, disease free survival (DFS) and overall survival (OS) was 25.1% (95% CI, 7.3−42.9%), 59% (95% CI, 33.3−84.7%) and 78.4% (95% CI, 63−93.8%), respectively. The CIs of CMV and EBV reactivation by day 180 were 37.5% (95% CI, 19.8−55.2%) and 40.6% (95% CI, 22.6−58.6%), respectively. The results suggested that low-dose ATG with low-dose PTCy as GvHD prophylaxis in Haplo-PBSCT-Cord had promising activity.

## Introduction

Acute graft-versus-host disease (aGvHD) is the most important obstacle of haploidentical hematopoietic stem cell transplantation (Haplo-HSCT) for treatment of patients with hematologic malignancies. In the last two decades, the results of Haplo-HSCT have been conspicuously improved due to effective prophylaxis strategies for aGvHD, such as in vivo T-cell depletion (TCD) with anti-thymocyte globulin (ATG) or posttransplant cyclophosphamide (PTCy) [1, 2].

The regimens for prophylaxis of GvHD based on rabbit anti-human thymocyte immunoglobin (ATG 10 mg/kg, Thymoglobin®, Genzyme Polyclonals S.A.S) effectively prevented the occurrence of grade II-IV aGvHD with incidences of 33.4−46%, grade III-IV aGvHD 12−14.9%, but the reactivation incidences of cytomegalovirus (CMV) and EB virus (EBV) were higher due to a slower immune reconstitution [2−4]. The 100-day CIs of CMV and EBV viremia were 61−64% [2, 5] and over 50% [5, 6], respectively. Although ATG-based regimens have achieved excellent results, the incidences of aGvHD and the posttransplant virus reactivation are still higher, affecting the long-term survival of the patients.

The regimen of PTCy for prevention of GvHD was developed in 1999 by St. Johns Hopkin’s group in Baltimore [1] and had outstanding results with the CIs of 34% grades II-IV and 6% grades III-IV aGvHD by day 200 in Haplo-bone marrow transplantation (Haplo-BMT) [7], respectively. Compared with standard PTCy regimen (two doses of PTCy; day +3, +4), one dose of PTCy (day +3) had similar effects in preventing aGvHD, but was less effective for chronic GvHD (cGvHD), especially serious cGvHD [7]. The incidences of viral and fungal infection in Haplo-HSCT with PTCy for GvHD prophylaxis were much lower than ATG-based regimens. Ruggeri et al. [8] retrospectively analyzed the effects of different stem cell source (BM *vs* peripheral blood stem cell (PBSC)) on transplant results in Haplo-HSCT with PTCy. The results showed that BM graft was associated with a lower incidence of grades II-IV and grades III-IV acute GVHD (21 *vs* 38%, *P* ≤ .01; and 4 *vs* 14%, *P* < .01, respectively), which was further confirmed by Bashey et al.’s study [9]. These data indicated that PTCy regimen did not have the same effects for GvHD prophylaxis with PBSC graft as compared with BM graft in Haplo-HSCT.

A novel regimen, which is composed of a low dose of ATG (5 mg/kg) and low-dose PTCy (one dose of PTCy, 50 mg/kg) for GvHD prophylaxis in Haploidentical peripheral blood stem cell transplantation combined with unrelated cord blood (Haplo-PBSCT-Cord) for patients with hematologic malignancies, was designed to decrease the risk of aGvHD and lower the incidence of virus reactivation. A prospective, phase II clinical trial (Clinicaltrials.org NCT03395860) was performed to evaluate the efficacy with low-dose ATG followed by low-dose PTCy as GvHD prophylaxis. The results suggested that the novel regimen had excellent outcomes for aGvHD prophylaxis in Haplo-PBSCT-Cord.

## Patients and methods

### Patients’ eligibility

Total 32 eligible patients were enrolled into the clinical trial with ages from 20- to 62-year-old with hematologic malignancies including acute myeloid leukemia (AML), acute lymphoblastic leukemia (ALL), high-risk myelodysplasia (MDS), chronic myelomonocytic leukemia (CMML) from June 2017 to January 2018 in our center. ECOG performance status was ≤2 in all patients. All patients had adequate baseline laboratory values for eligibility. This study had ethical approval from the local ethical committees and conducted in accordance with the Declaration of Helsinki. All patient data originate from clinical trials with mandatory written informed consent.

Patients were ineligible if they had a central nervous system (CNS) disease, active uncontrolled bacterial, viral or fungal infections, or severe organ dysfunction, including a left ventricular ejection fraction < 40% and alanine transaminase (ALT)/aspartate transaminase (AST) > 2.5× the upper normal limit. The comorbidity index of each patient was calculated using the hematopoietic cell transplantation-comorbidity index system.

### Study design and treatment

#### Donors and stem cell sources

Family members selected as donors were typed on the HLA-A, -B, -C, -DRB1 and -DQB1 locus at high-resolution level. Haplotype was defined as recipient-donor number of HLA mismatches > 3 [10]. Unrelated cord blood (UCB) was co-infused with PBSC graft as the third-party cells at 4 h before the stem cell graft infusion. UCB was preferentially selected based on HLA 4-5/6 matched to total cell number. The PBSC grafts for all patients were mobilized with G-CSF. The target value for CD34^+^ cells in mobilized PBSC graft is a minimum of 10×10^6^/kg of recipient weight.

#### Conditioning regimens

**Fig. 1:**
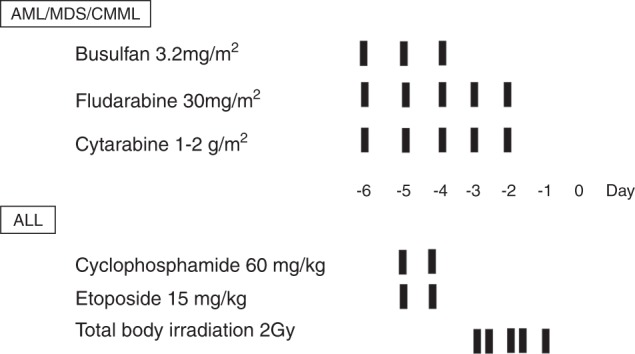
The schema of myeloablative conditioning (MAC) regimens for myeloid malignancies and acute lymphoblastic leukemia

Reduced-intensity conditioning (RIC) was given to the patients above 55 years old (≥55 years), while myeloablative conditioning regimens (MACs) were designed for patients below 55 years old (<55 years). The conditioning regimens for myeloid malignancies including AML, MDS and CMML were based on intravenous busulfan 3.2 mg/kg/day for 3 days in MACs (2 days in RIC), fludarabine 30 mg/m^2^/day and cytarabine (Ara-C) 1−2 g/m^2^/day for 5 days. All the patients with ALL received MAC regimen, which consisted of total body irradiation (10 Gy in five fractions), cyclophosphamide (60 mg/kg/day for 2 days) and etoposide (15 mg/kg/day for 2 days) (Fig. 1)

#### GvHD prophylaxis

The GvHD prophylaxis consisted of ATG 2.5 mg/kg administered on day −2 to −1 and cyclophosphamide (Cy) 50 mg/kg on day +3, cyclosporine A (CsA) and mycophenolate mofetil (MMF) initiating on day +4. CsA was prescribed at 2 mg/kg as a continuous infusion. The CsA doses were modified to obtain nadir serum levels between 200 and 300 ng/ml. MMF was administered at 15 mg/kg oral three times per day (maximum dose 3 g per day) until day +34 and was then stopped if no aGvHD. Mycophenolate Sodium Enteric-coated Tablets (MPA) can be used instead of MMF; one tablet MPA corresponds to one tablet MMF. CsA was tapered from day +90 to day +180.

#### Supportive care

G-CSF was given to all patients since day +5 until neutrophil recovery. All patients received prophylactic levofloxacin, acyclovir from the beginning of conditioning therapy until hematological reconstitution. Prophylactic posaconzale was administered from the day of the conditioning until 1 month after transplant [11]. Quantitative real-time PCR assays for CMV DNA in serum and EBV DNA in whole blood were performed once or twice per week. Preemptive therapy with ganciclovir (5 mg/kg, twice) started if CMV DNA was more than 1000 copies/ml. Preemptive therapy with rituximab (a single dose 375 mg/m^2^) was initiated when EBV DNA increased a log or more within 1 week or above 1×10^5^ copies/ml in high-risk patients [12] with EBV reactivation. EBV-related posttransplant lymphoproliferative disease (PTLD) was treated with reducing dose of immunosuppressive agents (RIS) and rituximab once per week for maximum of four courses [13].

#### Engraftment and GvHD evaluation

The engraftment endpoint was based on neutrophil engraftment with absolute neutrophil count (ANC) ≥ 0.5×10^9^/l for 3 consecutive days after transplantation without G-CSF. Platelet engraftment was defined as the first of 7 consecutive days with platelet counts of >20×10^9^/l without platelet transfusion. Graft failure was defined as failure of neutrophil engraftment on day 28 following transplantation (primary graft failure), or loss of donor chimerism after initial engraftment with ≥95% recipient cells at any time, not due to relapsed disease (secondary graft failure) [14].

aGvHD was diagnosed and graded according to the modified Glucksberg grading of aGvHD [15]. cGvHD was diagnosed and graded according to the 2014 National Institutes of Health (NIH) consensus criteria: mild, moderate, or severe, respectively [16].

#### Chimerism monitoring

Quantitative chimerism monitoring [17] was performed by short-tandem repeat (STR)-based PCR techniques on CD3-positive cell population from bone marrow and by fluorescent in situ hybridization [18] for patients with sex-mismatched donors at regular intervals of every 4 weeks after transplant within first 6 months. The chimerism of cord blood was also monitored after transplantation with STR-based PCR techniques until continuous twice results without cord blood components. Mixed T-cell chimerism was defined as between 5 and 94% recipient cells and complete donor chimerism was defined as the presence of more than 95% donor cells at all measured time points [19].

### Statistical analysis

The primary endpoint was the CIs of aGvHD within 100 days after Haplo-PBSCT. The secondary endpoint was the engraftment (leukocyte and platelet engraftment, donor chimerism), NRM, cGvHD, the CIs of relapse (CIRs), OS, disease-free survival (DFS), CIs of CMV viremia and CMV-associated diseases, EBV viremia and PTLDs. Only patients with successful ANC engraftment were evaluated for aGVHD and cGVHD was evaluated only in patients with a minimum follow-up of 100 days. CIR was calculated from the date of allo-HSCT or the date of getting CR after transplantation until relapse. The response criteria for CR and relapse were defined according to the literature [20]. Primary refractory disease was defined as the failure of achieving CR after two cycles of initial induction chemotherapy or hematologic relapse within 6 months from the beginning of initial therapy. Secondary refractory disease was defined as relapse from CR and had no response to salvage chemotherapy. NRM was defined as death without evidence of disease relapse. All statistical tests were two-sided and *P* value < 0.05 was considered significant. The statistical analyses were performed using IBM SPSS 17.0 statistical software (IBM, North Harbour, Portsmouth, UK).

## Results

### Patient characteristics

Thirty-two patients with hematologic malignancies were diagnosed and recruited from June 2017 to January 2018 in our center. Median age was 37 years (range, 20−62 years). At the time of transplantation, a total of 14 patients with AML/ALL reached first or subsequent complete response (CR1, ≥CR2) with conventional therapy or salvage therapy; the other 18 patients had active disease without responding to salvage therapy including three ALL, three MDS-EB II, two CMML and ten AML patients. The overall characteristics of the patients and donors are summarized in Table 1.

**Table 1:** Patient demographics

	Median (range)
Median age	37 (20-62)
Sex	
Male	18
Female	14
Median interval from diagnosis to	8 (4−60)
HSCT, months(range)	
Diagnosis	
De novo AML	22
ALL	5
MDS-EB II	3
CMML	2
Disease status at transplantation	
CR1	12
≥CR2	2
Primary refractory	7
Secondary refractory	11
HCT-CI Median (range)	0 (0−1)
Conditioning regimens	
MAC	29
RIC	3
Donor relationship with patients	
Sibling	7
Parents	8
Mother/father	
Offspring	15
Cousin	2

*AML* acute myelocytic leukemia, *MDS-EB II* myelodysplastic syndrome with excess blasts, type II, *CMML* chronic myelomonocytic leukemia, *ALL* acute lymphoblastic leukemia, *CR* complete remission, *MAC* myeloablative conditioning regimen, RIC reduced-intensity conditioning, *HCT-CI* Hematopoietic Cell Transplantation-Comorbidity Index [34]

### Engraftment

All patients received G-CSF mobilized PBSC with MNCs 15.5 (7.5−28.3)×10^8^/kg and CD34^+^ cells 13.39 (2.4−31.7)×10^6^/kg. The median nuclear cell number and CD34^+^ cell number of unrelated third-party cord blood were 2.1 (1.4−4.14)×10^7^/kg and 5.6 (1.52−11.2)×10^4^/kg, respectively. The median time for neutrophil engraftment was 12 days (range 9−20) for patients with primary engraftment; whereas the median time for platelet engraftment was observed in 16 days (range 12−25). The results of chimerism monitoring showed that all of these patients were fully donor chimerism when assessed between days 14 and 28 after transplantation. Primary graft failure occurred in one patient (3.1%) with CMML who received lower CD34^+^ cell number of 2.4×10^6^/kg. One patient with AML experienced secondary graft failure after CMV pneumonia at day 90. There was no chimerism of cord blood cells in all patients on day 14 after transplantation.

### Immune reconstitution

**Fig. 2:**
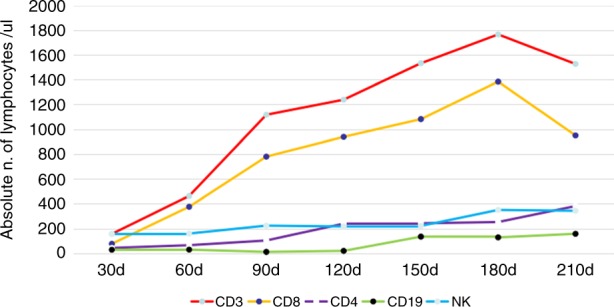
Immune reconstitution after transplantation (data were shown as median cell counts/μl)

The median lymphocyte counts, stratified by CD3^+^, CD4^+^, CD8^+^, CD19^+^ and CD56/CD16^+^, is depicted in Fig. 2. Twenty-two patients were included in immune reconstitution studies and 15 cases were analyzed at each endpoint. On day +100 and +120, median CD3^+^, CD4^+^ CD8^+^, CD19^+^ and CD56/CD16^+^ counts were 1117 (442−2136) and 1239 (494−2117), 104 (31−159) and 240 (201−487), 780 (310−1914) and 939 (366−1614), 12 (6−42) and 20 (10−145), 224 (78−1961) and 217 (64−1680)/μl, respectively. Since 120 days after transplantation, CD4^+^ cell counts were all above 200/μl and reached nearly 400/μl on +210 days.

### GvHD and infectious complications

**Fig. 3:**
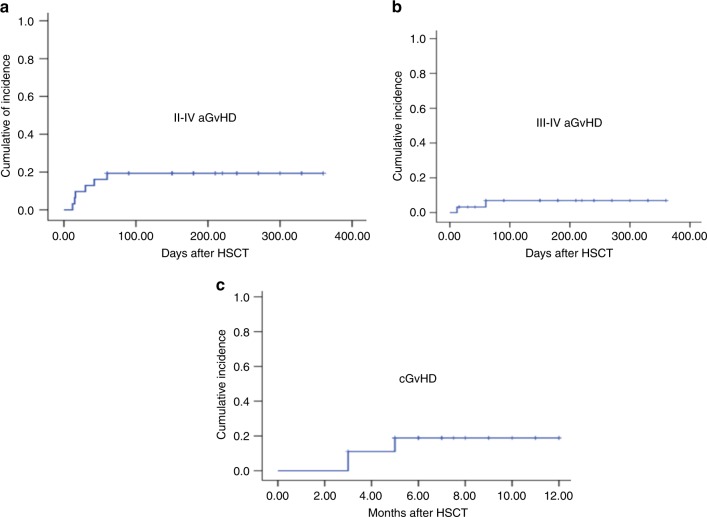
The cumulative incidences (CIs) of graft-versus-host disease (GvHD). **a** The CIs of grades II-IV aGvHD. **b** The CIs of grades III-IV aGvHD. **c** The 1-year CIs of cGvHD

The CI of grades II-IV aGvHD was 19.4% (95% CI, 5.5–33.3%) (Fig. 3a) and grades III-IV aGvHD was 6.9% (95% CI 0–16.3%) (Fig. 3b) within 100 days after transplantation. The frequencies of aGVHD at day +100 and +180 were same due to no patient suffering from late onset aGVHD. The grade II aGvHD involvement of isolated skin occurred in two patients, while skin and gut in two patients. The grade III aGvHD involvement of gut and skin occurred in one patient, while skin and liver in another patient. No patient developed grade IV aGvHD. All six cases with aGvHD received systemic corticosteroids (methylprednisolone (MP) with 2 mg/kg/d). Two of them required secondary treatment with anti-CD25 mAbs (basiliximab, Novartis Pharma AG, Basel, Switzerland) because of refractory to primary therapy and both got complete remission. The CIs of moderate-to-severe cGvHD within 6 months were 18.8% (5/27 cases, four with moderate cGVHD and one with severe cGVHD (95% CI, 3.933.7%)) (Fig. 3c). No patient died from acute and chronic GvHD. The CIs of CMV and EBV reactivation by day +180 were 37.5% (12 cases) (95% CI, 19.8−55.2%) and 40.6% (13 cases) (95% CI, 22.6−58.6%) respectively. Six patients suffered from pneumonia (bacterial pneumonia in one patient, aspergillus pneumonia in three patients, CMV pneumonia in one patient and pneumocystis carinii pneumonia in one patient). One patient was diagnosed as PTLD at 3 months after transplantation and died from aspergillus pneumonia (Table 2).

**Table 2:** Infectious complications after haplo-PBSCT-Cord

	*N* (%)
CMV viremia	12 (37.5%)
CMV disease	1 (3.1%)
EB infection viremia	13 (40.6%)
PTLD EB related	1 (3.4%)
Hemorrhagic cystitis BK virus-related	5 (15.6%)
Pneumonia	
Virus	1 (3.1%)
Fungal	3 (9.4%)
Bacteria	1 (3.1%)
Pneumocystis carinii pneumonia	1 (3.1%)

*CMV* cytomegalovirus, *PTLD* posttransplant lymphoproliferative disorder

### NRM, relapse and survival

Three patients died of nonrelapse causes (NRM 3/32, 9.4 %). One patient with CMML in non-remission (NR) experienced primary graft failure due to a low number of CD34^+^ cells in graft from his 60-year-old donor and finally died from CNS hemorrhage at 1.5 months after transplantation. One patient with AML experienced secondary graft failure after CMV pneumonia and soon died. Another patient died from aspergillus pneumonia at 6 months after transplantation (Table 3). There was no death due to acute or chronic GvHD.

**Table 3:** Causes of death

Causes of death	No.
Relapse	3
GvHD	0
Lung infection	2
CNS hemorrhage	1

*GvHD* graft-versus-host disease *CNS* central nervous system

**Fig. 4:**
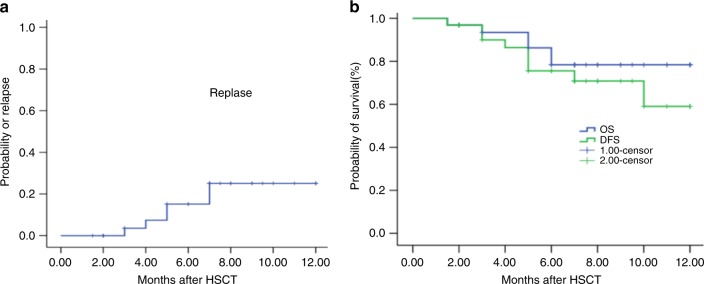
Clinical outcomes after Haplo-PBSCT-Cord. **a** The 1-year probability of relapse. **b** The 1-year probability of disease-free survival (DFS) and overall survival (OS)

Seventeen out of 18 patients with active disease achieved CR after transplantation. With a median follow-up of 7 months (range, 2−12 months), six cases relapsed. All the six patients were in disease active status with blasts from 6 to 18% at the time of transplantation. Four of five patients with normal cytogenetics had DNMT3-A mutation (one case), NRAS mutation (two cases), and MLL-AF9 rearrangement (one case), respectively. One patient with ALL had cytogenetic abnormalities with 46xy, der(3), der(9). The median time of relapse was 4.5 (3−10) months. One patient achieved the second CR (CR2) and was still alive after chemotherapy followed by donor lymphocyte infusion (DLI) for 5 months. Two patients were still in treatment. All other three patients died from relapse without treatment (Table 3). The 1-year probability of relapse was 25.1% (95% CI, 7.3−42.9%) for all patients (Fig. 4a). The 1-year probabilities of DFS and OS were 59% (95% CI, 33.3−84.7%) and 78.4% (95% CI, 63−93.8%), respectively (Fig. 4b).

## Discussion

Data from our prospective study showed that the CIs of grades II-IV aGvHD and grades III-IV aGvHD within days 100 posttransplantation were 19.4% (95% CI, 5.5–33.3%) and 6.9% (95% CI 0–16.3%) in Haplo-PBSCT-Cord with low-dose ATG plus low-dose PTCy regimen for GvHD prophylaxis respectively, which were much lower than the results using PTCy regimen or ATG-based regimens [2, 7]. The incidence of cGvHD was 18.8% (95% CI, 3.9−33.7%). No patient died due to aGvHD. The results suggested that low-dose ATG plus low-dose PTCy could effectively prevent aGvHD in patients who undergo Haplo-PBSCT-Cord as good as standard PTCy regimen for Haplo-BMT. PBSC graft was the independent risk factor of aGvHD in Haplo-HSCT with PTCy regimen [8]. We speculated that more T lymphocytes and different T-cell dynamics in PBSC graft post-transfusion as compared with BM graft might be the major factor for unsatisfied prevention of aGvHD with standard PTCy regimen for Haplo-PBSCT. T lymphocytes in G-CSF-primed PBSC graft might enter into cell cycle at an earlier time than that in BM graft after infusion, so that the first dose of Cy on day +3 in standard PTCy regimen might be late for early active T lymphocytes, because Cy could induce immune tolerance only when it was given precisely between 48 and 72 h, not 24 or 96 h after transplantation [21]. But it needs to be further studied. Pre-transfusion low-dose ATG could deplete early active T lymphocytes to prevent the occurrence of aGvHD in combination with PTCy on day +3 with synergistic effects due to their different mechanisms of ATG and Cy on T lymphocytes.

The engraftment rate was analyzed as the second clinical endpoint. All patients but one were successfully engrafted after transplantation. Thirty-one patients achieved full donor chimerisms between days 14 and 28 after transplantation. But one patient experienced secondary graft failure after CMV pneumonia on +90 days. The engraftment rate in our study was acceptable. In an earlier study about PTCy regimen for GvHD prophylaxis with NMAC regimen, the engraftment failure rate was 13% and it was improved by adding total dose of Cy 29 mg/kg on days –6 and −5 pre-transfusion, which suggested that increasing immunosuppressive intensity facilitated engraftment. Wang et al. [22] reported a randomized clinical trial comparing two different doses of ATG (6 and 10 mg/kg) as GvHD prophylaxis for Haplo-HSCT. There was no difference in the median myeloid and platelet engraftment time and the rate of graft failure. We speculated that the addition of low dose of ATG (5 mg/kg) at pretransfusion would ensure the engraftment. High dose of CD34^+^ cells in PBSC graft might be one reason to improve the engraftment rate in our study. In our study, 29/32 cases received MAC regimens, which was another reason for a higher engraftment rate. Solomon et al. [23, 24] reported that 100% of engraftment rate was achieved when MAC was given to patients of Haplo-HSCT with PTCy regimen for GvHD prophylaxis.

Mortality from GvHD and infection accounted for the vast majority of NRM in Haplo-HSCT. In our study, NRM was 9.4%, which was comparable to Haplo-HSCT with PTCy regimen and significantly lower than ATG-based GvHD prophylaxis regimens. No patient died from GvHD and only two patients died from infection. Because of the poor immunologic reconstitution, the rate of susceptibility to infection in Haplo-HSCT is higher than HLA-matched HSCT, especially with ATG-based regimens. Tischer et al. [5] retrospectively compared the incidence of virus infections and outcomes in the context of immune reconstitution between PTCy and ATG-based regimens. The results showed that the incidence of herpes virus infection was markedly lower in the PTCy group (22%) than in the ATG group (93%) and the recovery of CD4^+^ T cells on day +100 was faster in the PTCy group. CMV reactivation was also lower in the PTCy group than in the ATG group (30 *vs* 57%), virus infection-related mortality (VIRM) was significantly lower in the PTCy group (1-year VIRM, 0% PTCy *vs* 29% ATG; *p* = 0.009). Twenty-five percent of the patients in the ATG group but no patient in the PTCy group developed PTLD. In our study, median CD4^+^ lymphocyte counts were 104/μl on day +100, which were similar with ATG-based regimens (102/μl) and lower than with PTCy (206/μl) at the same time, but on day +120, CD4+ lymphocyte counts with 240/μl reached the level on day +100 in the PTCy group and higher than that (157/μl) on+180 days in the ATG-based group [5]. These data might imply that immune recovery was faster in our study than with ATG-based regimens and just a little slower than with PTCy regimen. The CIs of CMV and EBV reactivation by day +180 were 37.5% (95% CI, 19.8−55.2%) and 40.6% (95% CI, 22.6−58.6%), respectively, which were lower than with ATG-based regimens and comparable to the PTCy regimen.

Disease relapse remains a significant cause of treatment failure after allogeneic HSCT, especially for patients with active disease. In the present study, six cases relapsed and three of them died. All the six patients were in active disease. One patient was primary refractory with DNMT3-A mutation. The other five patients had no response to salvage chemotherapy. In previous reports [25, 26], PTCy-based regimens did not impact the relapse of hematologic malignancies after Haplo-HSCT as compared with HLA-matched transplantation and with different preconditioning intensity. In a recent report [27] grade II aGvHD could reduce relapse risk and improve the survival of patients with hematological malignancies with PTCy regimen for prophylaxis of GvHD in Haplo-BMT. In the study, all patients received NMAC regimens and all of the grafts were from bone marrow. But in our study, nearly all patients received MAC regimens and all of the grafts were from PBSC. These differences might lessen the effect of mild aGvHD on disease recurrence. The lower NRM (9.4%) in this study needs to be carefully interpreted, because of younger age, shorter follow-up and lower HCT score (0−1) of patients. The impact of our novel regimen of GvHD prophylaxis on relapse and NRM with longer follow-up and more samples needs to be further evaluated.

Haploidentical and unrelated cord blood (HID and UCB) are alternative donors for transplant patients without HLA-matched related or unrelated donors. The different combination strategies of haploidentical graft and UCB were successfully used to treat patients with hematological disease. The Spanish’s group [28, 29] and University of Chicago from America [30, 31] reported that the UCB cells in combination with CD34^+^ selected PBSCs from a related mismatched donor were transplanted to patients with fast engraftment, low incidences of GvHD, and promising long-term results. Tsai et al. [31] reported that haplo/cord reduced-intensity transplantation followed by durable cord blood engraftment achieved similar outcomes compared with HLA-matched unrelated donor (MUD) in older AML and MDS patients. A group from China [32, 33] evaluated another combination strategy of un-manipulated haploidentical stem cells combined with a small dose of umbilical cord blood from a third-party donor (haplo-cord transplant) for hematological malignancies, which was followed by durable haploidentical stem cell engraftment. The results showed that haplo-cord transplant may potentially improve the outcome of HID- and UCB-HSCT alone. To improve the outcomes of haploidentical transplantation, the second combination strategy of un-manipulated haploidentical PBSCs with an UCB (Haplo-PBSCT-Cord) was adopted in our study.

In conclusion, this is a pilot study to establish a low-dose ATG with low-dose PTCy as an effective regimen to prevent GvHD in Haplo-PBSCT with a lower risk of virus reactivation and a higher engraftment rate. But this study still had several limitations, including a lower number of samples, a shorter follow-up, the combination of haplo PBSCs with UCB, etc. A prospective randomized trial without UCB co-infused is required to compare the efficacies of this regimen with ATG-based or PTCy-based regiments in Haplo-PBSCT.
